# Mitochondrial diabetes presenting with spontaneous abortion and ketoacidosis onset: A case report and literature review

**DOI:** 10.1097/MD.0000000000040039

**Published:** 2024-10-18

**Authors:** Baoyuan Wu, Yubing Tao, Qingqiang Wu, Caiyan Zou, Xuekui Liu, Houfa Geng

**Affiliations:** a Medical Science and Technology Innovation Center, Shandong First Medical University and Shandong Academy of Medical Sciences, Jinan, China; b Department of Endocrinology, Xuzhou Central Hospital, Xuzhou, China.

**Keywords:** diabetic ketoacidosis, genetic mutation, mitochondrial diabetes, spontaneous abortion in pregnancy

## Abstract

**Rationale:**

Mitochondrial diabetes mellitus (MDM) is a rare form of diabetes characterized by mitochondrial dysfunction, leading to a diverse range of clinical manifestations that may result in misdiagnosis. Accurate identification of MDM is essential for proper management and reporting of diagnosed cases.

**Patient concerns:**

The patient was a young woman with a slender physique who presented with sensorineural hearing loss detected during auditory testing.

**Diagnoses:**

Auditory testing confirmed sensorineural hearing loss, and further evaluation revealed impaired pancreatic β-cell function, indicating reduced insulin secretion. Genetic testing of blood samples identified the A3243G mitochondrial DNA mutation. The patient’s family history was notable for hearing loss in her mother and maternal grandmother, who exhibited clinical features consistent with MDM.

**Interventions:**

Clinical management focused on monitoring and addressing the metabolic and clinical needs associated with MDM.

**Outcomes:**

The diagnosis of MDM was established, highlighting the importance of recognizing the diverse clinical manifestations, including a rare case of spontaneous abortion during pregnancy.

**Lessons:**

MDM presents with atypical clinical manifestations, and thorough physical examinations are crucial for its diagnosis. This case underscores the significance of genetic testing and family history in diagnosing MDM and the need for increased awareness among clinicians to prevent misdiagnosis.

## 1. Introduction

Mitochondrial diabetes mellitus (MDM) is a rare type of diabetes caused by genetic mutations that result in mitochondrial dysfunction.^[1]^ It is relatively uncommon in clinical practice, with some cases presenting with spontaneous abortion in pregnancy and subsequent diabetic ketoacidosis.^[2]^ This report describes a patient hospitalized in October 2023 due to spontaneous abortion in late pregnancy. The case was complicated by diabetic ketoacidosis and was ultimately diagnosed as MDM. Furthermore, there is limited available information on this condition in domestic and international research. Hence, we analyzed the clinical characteristics, diagnostic process, and treatment of the case. A review of relevant literature is also presented, aiming to assist clinicians with the identification, diagnosis, and therapeutic approaches associated with MDM.

## 2. Case report

A 33-year-old pregnant woman presented at the Xu Zhou Maternal and Child Health Hospital with paroxysmal abdominal pain. The pregnancy was at 26 weeks and 2 days gestation, and she miscarried. Laboratory examination revealed elevated random blood glucose (30.7 mmol/L), urine glucose (++++), and urine ketones (+++). Blood gas analysis confirmed diabetic ketoacidosis with a pH of 7.31. Despite aggressive hydration and intravenous insulin therapy, there was no improvement in her condition, leading to a referral to the Endocrinology Department of Xu Zhou Central Hospital for further management. The patient reported to have experienced polydipsia, polyuria, and fatigue 1 month before admission. However, she had no neurological symptoms, such as blurred vision, numbness, or limb convulsions. Notably, she reported to have experienced progressive hearing loss since 2017. Her menstrual history indicated that she had had regular cycles since menarche at the age of 13, occurring every 30 days with a duration of 5 days. Importantly, she had previously given birth after natural conception and had 1 child with a birth weight of 3.0 kg.

Family history: Both the patient’s mother and maternal grandmother had a history of sensorineural hearing loss.

Physical examination: The patient presented with clear consciousness with fluent speech and cooperation during the examination, although mental acuity was diminished. Vital signs were within normal ranges, with a temperature of 36.7°C, blood pressure of 107/110 mm Hg, and a pulse rate of 79 beats/min. She had a short stature with a height of 142 cm and a weight of 32.0 kg, with a body mass index (BMI) of 16.3 kg/m^2^. There was no evidence of abnormal hair distribution, and thyroid palpation revealed no bilateral enlargement. Mild dehydration was evident, while no Cushingoid features were observed. Further, cardiovascular, respiratory, and abdominal examinations displayed no abnormalities. Tanner staging of the external genitalia showed a classification of P5B5. Sensory examination of the extremities indicated normal function, with muscle strength of grade V and an absence of pathological reflexes.

Further relevant investigations revealed fasting blood glucose levels of 14.9 mmol/L and a glycated hemoglobin level of 10.6%, with a glycated albumin level of 30.72%. Electrolyte levels were within normal range, with potassium at 3.89 mmol/L, sodium at 138.4 mmol/L, and chloride at 109 mmol/L. Additionally, the lipid profile showed 4.56 mmol/L triglycerides, 4.01 mmol/L total cholesterol, 1.01 mmol/L high-density lipoprotein cholesterol, and 1.38 mmol/L low-density lipoprotein cholesterol. Enzyme level analyses revealed lactate dehydrogenase at 333.8 U/L, hydroxybutyrate dehydrogenase at 282.1 U/L, creatine kinase at 83 U/L, and creatine kinase isoenzymes at 34.5 U/L. The urine analysis showed glucose++, ketones+, protein+, and a high urine albumin/creatinine ratio. Blood gas analysis revealed a pH of 7.374, carbon dioxide partial pressure of 27.4 mm Hg, and lactate at 2.7 mmol/L. Pancreatic autoantibodies (islet cell antibodies, glutamic acid decarboxylase antibodies, and insulin antibodies) were not detected. Insulin-release tests indicated impaired function, with a fasting insulin level in the serum of 0.26 µU/mL, a 2-hour insulin measurement of 0.69 µU/mL, a fasting C-peptide serum concentration of 0.56 ng/mL, and a 2-hour C-peptide level of 0.90 ng/mL. An electrocardiogram showed sinus rhythm, while echocardiography revealed trivial aortic valve regurgitation. No abnormalities were observed in vascular Doppler ultrasound of the lower limbs and nerve conduction studies. Pure-tone audiometry and impedance testing indicated bilateral sensorineural hearing loss.

**Figure 1.**
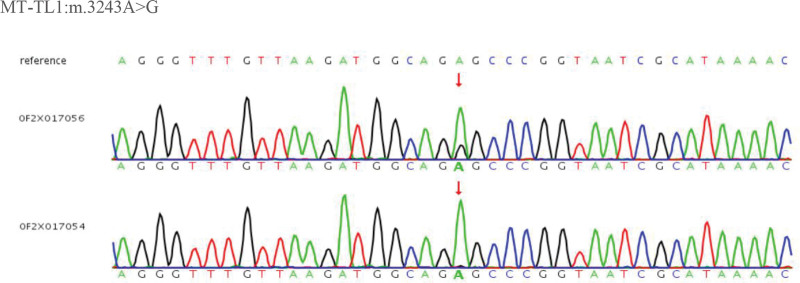
Single nucleotide variants and InDels.

Genetic testing: Whole-exome sequencing for mitochondrial genetic mutations was performed on whole-blood samples at Beijing Berry and Kang Medical Laboratory. The results revealed a heterozygous mutation of mtDNA A3243G with 27.8% mutation rate. Tests for mitochondrial genetic mutations in blood samples from the patient’s mother was negative (Fig. 1). Genetic testing of other relatives of the patient has not yet been conducted.

## 3. Results

The patient was diagnosed with MDM, diabetic ketoacidosis, and late-stage spontaneous abortion based on the clinical characteristics and genetic testing results. Treatment Plan: continuous subcutaneous infusion of insulin using an insulin pump was initiated initially, followed by basal-bolus insulin therapy with Degludec and NovoRapid (10 U before breakfast, 4 U before dinner) after achieving glycemic control. The patient was followed up 3 months after discharge, and the treatment plan was modified to Degludec and NovoRapid (8 U before breakfast, 4 U post-dinner) as peripheral blood glucose monitoring showed fasting levels of 5 to 7 mmol/L and postprandial levels of 8 to 10 mmol/L. No further deterioration in hearing loss was observed during follow-up. The results of partial blood biochemical indices and pancreatic function tests pre and posttreatment are shown in Table 1.

**Table 1:** Results of partial blood biochemical indices and pancreatic function tests before and after treatment of the patient.

	Glu (mmol/L)		HbA1c (%)	C-P (ng/mL)	
	0 h	2 h		0 h	2 h
On admission	14.9	27.1	10.6	0.56	0.90
3 mo after discharge	5.7	8.5	7.7	0.64	1.27

Note: C-P: c-peptide, Glu: glucose; HbA1c: glycosylated hemoglobin type A1c.

## 4. Discussion

Mitochondria are the only organelles in the cell, other than the nucleus, that carry genetic material. They contain genes for 2 rRNAs and 22 tRNAs involved in the synthesis of respiratory chain complexes.^[3]^ Mitochondrial diseases are relatively rare in clinical practice and are caused by mutations in mitochondrial DNA.^[4]^ Mitochondrial oxidative phosphorylation to produce triphosadenine (ATP) is 1 of the main sources of energy in eukaryotic cells.^[5]^ However, mitochondrial DNA is less efficient in self-repair due to the lack of introns in its genome and the absence of histone protection, making it more prone to mutations. These mutations can lead to mitochondrial dysfunction that leads to an inability to produce sufficient ATP.^[6]^ Multiple organ dysfunctions may occur due to the strong heterogeneity of mitochondrial diseases. These mainly affect metabolically active tissues such as bone, nerves, muscles, pancreas, kidneys, and inner ears.^[1–3]^ Therefore, the symptoms of mitochondrial diseases may manifest in multiple organ systems.

MDM, also known as maternally inherited diabetes and deafness syndrome (MIDD), is a mitochondrial disease often characterized by maternal inheritance and sensorineural deafness.^[7]^ This syndrome was first described by Van den Ouweland^[8]^ and Reardon.^[9]^ It was first reported in 1995 in China by Kunsan Xiang.^[10]^ Research suggests that close to 1% of young patients with diabetes have mitochondrial diabetes.^[7]^ Statistics show that about 85%^[11]^ of mitochondrial diabetes cases are associated with the mtDNA A3243G mutation. There are also several less common mutation sites, such as mtDNA.G9267C, mtDNA.T14530C,^[12]^ and mtDNA.A09155G.^[13]^ The heterogeneity of mitochondrial genetic mutations in mitochondrial diabetes is also associated with the severity of clinical manifestations.^[14,15]^ Different degrees of mutations in the mtDNA can lead to varying clinical manifestations. For instance, a low mutation rate (<40%) of mtDNA A3243G in cells may manifest as mitochondrial diabetes or sensorineural deafness. However, at a higher mutation rate (>70%), there can be more severe clinical symptoms, such as cardiac damage or cerebral attacks.

The pathogenesis of diabetes induced by mitochondrial mutations is not yet fully understood. Given that substances, such as ATP produced by mitochondria are involved in the insulin secretion process, mitochondria play a crucial role in insulin secretion. Mutations can disturb cellular oxidative phosphorylation or induce an increase in toxic OH- substances, causing DNA damage and reducing ATP synthesis, resulting in insufficient energy generation. This can lead to the closure of ATP-dependent potassium channels and the opening of calcium channels, thus affecting insulin vesicle exocytosis and reducing insulin secretion.^[1]^ Furthermore, mutations in mitochondrial superoxide dismutase and increased levels of reactive oxygen species in mitochondria can lead to increased levels of pancreatic β-cell apoptosis.^[16]^

The patient in this report was a 33-year-old woman with short stature, slender physique, and sensorineural deafness. The results of the pancreatic autoantibody test were negative, and the insulin-release test indicated low insulin secretion. Abnormal levels of myocardial enzymes in the blood were observed, along with ketoacidosis, hyperlactatemia, and an elevated urinary microalbumin/creatinine ratio. High-throughput genetic sequencing of whole-blood revealed the presence of the mtDNA A3243G mutation, consistent with the diagnosis of MDM. Although genetic sequencing of the mother’s blood did not identify any abnormalities, she also had sensorineural deafness, as did the patient’s maternal grandmother. As leukocytes have a higher turnover rate the proportion of the mtDNA A3243G mutation in leukocytes from patients with MDM typically < 40%,^[17]^ and decreases with age. Conversely, compared to blood samples, tissues with lower turnover rates, such as muscle and urinary sediment cells, show relatively stable mutation rates, resulting in a higher rate of detection.^[18]^ Given the relatively low positivity rate of blood samples of older individuals, the patient’s familial genetic predisposition needs to be further confirmed through multiple tests on urinary sediment, muscle tissue, and other specimens from her mother and other relatives.

The patient was also diagnosed with blood sugar abnormalities in late pregnancy, which coincided with spontaneous abortion and subsequent ketoacidosis, resulting in the diagnosis of MDM. There are few reports of similar cases in either the domestic or international literature. On 1 hand, apart from MDM, increases in the levels of insulin-resistant substances during pregnancy reduce insulin sensitivity, resulting in elevated blood sugar levels. On the other hand, significant disruptions in glucose metabolism can significantly raise the likelihood of both miscarriage and fetal malformation in pregnant patients. This highlights the need for improved screening for diabetes in pregnant women. The early detection of gestational diabetes and its timely treatment are crucial for reducing the chances of miscarriage and fetal malformation. Specifically, young women with characteristics such as short stature, slender physique, low BMI, sensorineural deafness, or family history, together with negative results on tests for pancreatic autoantibodies and low insulin secretion should undergo early genetic sequencing. If necessary, testing of sensitive tissues, such as muscle tissue and urinary sediment cells, should be performed to facilitate both early detection and treatment of MDM. This approach would lead to improved quality of life and reproductive outcomes for affected patients.

## Acknowledgments

We acknowledge and thank the patient for her cooperation and sample contributions.

## Author contributions

**Conceptualization:** Xuekui Liu.

**Data curation:** Yubing Tao, Qingqiang Wu, Caiyan Zou, Xuekui Liu.

**Resources:** Qingqiang Wu, Houfa Geng.

**Validation:** Caiyan Zou.

**Writing – original draft:** Baoyuan Wu, Houfa Geng.

**Writing – review & editing:** Houfa Geng.
